# Validation Through Collaboration: Encouraging Team Efforts to Ensure Internal and External Validity of Computational Models of Biochemical Pathways

**DOI:** 10.1007/s12021-022-09584-5

**Published:** 2022-05-11

**Authors:** Richard Fitzpatrick, Melanie I. Stefan

**Affiliations:** 1 Centre for Discovery Brain Sciences, University of Edinburgh, Edinburgh, UK; 2 ZJU-UoE Institute, Zhejiang University, Haining, China; 3 School of Biological Sciences, University of Edinburgh, Edinburgh, UK

**Keywords:** Systems biology, Reproducibility, Computational modelling, Model validity, 92-08, 92C20, 92C42, 92C45

## Abstract

Computational modelling of biochemical reaction pathways is an increasingly important part of neuroscience research. In order to be useful, computational models need to be valid in two senses: First, they need to be consistent with experimental data and able to make testable predictions (external validity). Second, they need to be internally consistent and independently reproducible (internal validity). Here, we discuss both types of validity and provide a brief overview of tools and technologies used to ensure they are met. We also suggest the introduction of new collaborative technologies to ensure model validity: an incentivised experimental database for external validity and reproducibility audits for internal validity. Both rely on FAIR principles and on collaborative science practices.

## Introduction

The large number of molecules and interactions underpinning most biological phenomena call for in silico approaches to understand biochemical networks (Pollard 2013). This is especially true for neuroscience, where the interpretation of a molecular signalling network can have major implications on translational approaches to diseases and disorders. A good computational model makes testable predictions which can be used to narrow down the number of experimental investigations required to reach an understanding of a given phenomenon (Berro, 2018). With the newer multistate computational models and tools (Bazzazi et al., 2018; Boutillier et al., 2018; Harris et al., 2016; Stefan et al., 2014; Stites et al., 2015; Stefan et al., 2012; Pharris et al., 2019) we can see the impact of modifying one aspect of a molecule’s function on all others, and how that affects the biochemical network as a whole, without needing to construct many different computational models, or run multiple in vivo or in vitro experiments.

These powerful aspects of modelling have not reached their full potential within neuroscience. This may stem in part from a lack of clarity on how our modelling approaches represent the biological mechanisms they claim to simulate (Berro, 2018; Mogilner et al., 2006) and the soundness of the models themselves. Two major questions can be asked about the validity of a computational model:

1. How can we be sure that the model is representative of in vivo states?
2. How do we know the model is reliable?

The first question relates to the external validity of a model (how well the model fits with experimentally knowable data), the second relates to its internal validity (whether the model is soundly and consistently constructed).

This commentary provides two possible pathways to answer these questions about model validity. Both require a greater level of collaboration, both between biochemical modellers and between modellers and their experimental counterparts. We believe by fostering such connections models will be better utilised, better parameterised, and embedded more into the driving of neuroscientific inquiry.

## External Validity: Comparing a Computational Model to Experimental Data

Computational models of biological systems are important tools: They can synthesise the current state of knowledge about a biological process into a coherent system. Using models, we can explore overall behaviours of a biological system that would be impossible to predict from just examining its component parts (Le Novère, 2015). Using computational models, we can quickly test a large number of possible scenarios. They are therefore especially useful for generating hypotheses about a system, and making testable predictions about its behaviour.

The predictive power of a computational model relies on the model being an accurate (enough) representation of biological reality. Modellers rely on experimental data to construct and constrain the model. Once a model is completed, experiments are needed to validate models, test model predictions, or select from competing models of the same process.

A biochemical modeller wants their model parameters to closely resemble the situation in vivo. This requires binding constants and concentrations specific to a specific cell type or functional component such as a dendritic spine. There are databases of biochemical parameters (Glont et al., 2020; Jeske et al., 2019; Sivakumaran et al., 2003; Wittig et al., 2012), but at this point they suffer from incomplete coverage, especially when it comes to data on signalling pathways. For instance, in one of our models of CaMKII activation (Stefan et al., 2012), only $27\,\%$ of the model parameters were taken directly from experimental papers, another $13\,\%$ came from previous modelling papers, $27\,\%$ were derived from measurements found in the literature, and the rest ($33\,\%$) has to be estimated in the course of model construction and validation.

Compounding this data scarcity is that much of these experimentally derived sources for reaction constants and concentrations come from decades-old research. This work is often of excellent quality, but does not cover many more recently discovered molecules and interactions. The urgent need for new experimental data for models is being approached in interesting ways, with frameworks such as FindSim (Viswan et al., 2018) encouraging the integration of multiscale models with experimental datasets, and the FAIR initiative improving the extraction of data from published studies to improve discovery, standardisation, and enable the re-use of this data (Wilkinson et al., 2016). These cannot generate data which is not there though. As our in vivo techniques have improved, there has been a major shift in analysing the function of molecules in situ. The actual mechanisms that underpin function are often included quite late in how we currently construct and perceive biological theory (Lazebnik, 2002; Kennedy, 2017). This contrasts with biochemical modellers, who are almost always concerned with mechanisms (Chen et al., 2010; van Riel, 2006).

Modellers do have an array of tools to work around this problem. We can run parameter sensitivity analyses to pinpoint the parameters that matter to a reaction network (Zi et al., 2008), and then estimate values that fit with experimental outcomes. We can assess the robustness a reaction network, determining sloppy parameters, whose ’true’ value does not matter much to the behaviour of the model (Gutenkunst et al., 2007). Indeed it may be enough to focus experimental efforts on a few parameters that the model is most sensitive to, instead of measuring every single model parameter, which would be both experimentally costly and, for some parameters, unnecessary (Gutenkunst et al., 2007; Transtrum et al., 2015).

And yet, as we move to larger and more complex models, the question of how well these sensitivity and parameter identification analyses scale to larger contexts is still open (Babtie and Stumpf, 2017). Parameterisation techniques for large models have evolved rapidly to reduce the intractable computational load and to accommodate the sparsity of absolute datasets (Schmiester et al., 2020), but they do not eliminate the need to experimentally determine some key parameters.

Once built, a model can be tested against experimental data to establish its validity. A good model can make predictions that go beyond the currently available data, and may even go beyond currently possible experimental techniques - this is indeed quite common in fields such as physics (see for instance Englert & Brout, 1964; Higgs, 1964; Guralnik et al., 1964; ATLAS Collaboration, 2012; CMS Collaboration, 2012). This means that a modeller may predict the outcome of an experiment, but may never be able to conduct the experiment itself, or even see it conducted.

Taken together, experiments can provide useful information for computational modelling, from early on in model development to years after a model has been completed and distributed. For a fast-paced field with rich data such as neuroscience, where molecular understanding is constantly evolving, the ability to test hypotheses quickly and robustly against prior evidence is a valuable asset that modelling affords. There is a risk that a lack of biologically acquired parameters decouples modellers and experimenters. This causes work to be unnecessarily duplicated, or unfeasible avenues of research undertaken that could have been shown to be unwise with a single simulation or a simple in vivo test

We suspect all modellers have a “wish list” of experiments that would improve and accelerate their model development, or test model predictions. But not all modellers have access to the necessary infrastructure and skills. Finding experimental collaborators is not always easy: There is not necessarily complete overlap between the experiments that would be informative to a computational modeller and the experiments that interest the experimentalist.

## Incentivised Experimental Database

As we have seen, there is a gap between computational models and the experimental data needed to both constrain those models and test their outcomes. This is partly because existing data is not always published and shared, but partly also because some experiments have just never been conducted.

What is needed is a way of incentivising these experiments, to persuade our experimentalist colleagues that there is some benefit to them for carrying them out. We propose one such way is to take some lessons from the past. Could we not present our experimental wishlist, specifying the data we need to complete or check our models, and offer a cash incentive for providing this data?

Offering cash rewards for solving scientific problems is not without precedent. Historically, Challenge Prizes drove major advancements in problems of navigation (e.g. The UK Longitude Act of 1714 lon, 1714) and aviation (e.g the Orteig Prize Brady, 2002). The Millennium Prize offers $1 million for the solution of any of seven stated mathematical problems (Jaffe, 2006). Even more recently, foundations such as Nesta offer considerable sums of money for the solving of defined problems within a range of different fields (Puttick et al., 2014).

What we are suggesting is not quite financially on the same scale as this but captures some of that spirit; of incentivising innovation to accelerate improvements in biochemical modelling. Our “problem” is that we have limited access to specific biochemical data necessary to accelerate the construction and testing of complex dynamical models of biochemical systems. Our solution is to reward experimenters who “solve” parts of this problem through the provision of this data.

We envisage this working thusly. Modellers submit a wishlist of experiments to a database, with explicit instructions on the biological background, the model, the data needed, and (if available) a suggested experimental design. These experiments are sorted into categories related to difficultly and the experimental methodology required to implement them. The relative difficulty or complexity of the experiment is linked to a cash reward, which not only compensates for the time and resources used but provides extra income for the lab to continue their own research. These “microgrants” would be split into two components – money up front for the experiment, with the bonus provided upon submission of raw data and documentation following FAIR principles (regardless of the nature of the outcome). The dataset publication would also include authorship and contribution information of all experimental collaborators, as well as a link to the original data request and the model it stemmed from, thus giving credit and facilitating provenance tracking for model parameters.

These ‘arranged’ collaborations may even prove more fruitful in connecting researchers exploring the same phenomena through different approaches. This leads to our second parallel intention for this database: prediction testing.

As initiatives like FindSim (Viswan et al., 2018) emphasise, models ideally exist in a dynamic cycle with experimental research. Model outcomes produce predictions that are tested experimentally, which provides data to update the model to drive further predictions. This ideal scenario is rarely achieved however, and what we have is a mostly decoupled system where model predictions are not seen by experimental researchers, or only discovered after convergently reaching the same outcome. Our database would encourage modellers to post the major predictions of their models which can then be validated by experimental work. This approach allows for a gradual and visible increase in the utility of modelling alongside experimental work. As models receive higher fidelity parameter sets, the predictions made will have more weight and power to guide real conceptual breakthroughs.

This provides another use of the modeller “wish list”, supplying experimenters with readily available predictions and a clearly defined direction for potentially fruitful future research. The results of these investigations are importantly just as valuable if they contradict the model as when they agree, leading in each case to publishable findings and model enhancement.

Thus, the incentivised experiment database provides a mechanism for long-term mutually beneficial cycles of models and experiments to arrive at a deeper understanding of biological questions. Importantly, the cycle does not involve fixed teams of researchers. At any point, another experimentalist can claim an open experiment and contribute their data. And a modeller can pick up and refine an existing model. This brings us to the second issue around model validity: The “internal validity” of a computational model, i.e. its ability to be reproduced.

## Internal Validity: Ensuring Reproducibility of Computational Model

The importance of reproducible research has received much attention in recent years across all areas of science (Baker, 2016), including computational modelling of biological systems (Mendes, 2018). Reproducibility is an important condition for model sharing and reuse (Cucurull-Sanchez et al., 2019; Scharm et al., 2018).

Much work has been done on how to ensure the reproducibility of computational work. Standards for reproducible computational research have been formulated both as stand-alone guidelines (Sandve et al., 2013; Elofsson et al., 2019), and within the FAIR framework (Wilkinson et al., 2016).

Community efforts to ensure reproducible modelling of biochemical reaction systems include efforts to standardise model specification (e.g. Hucka et al., 2003; Zhang et al., 2020; Hedley et al., 2001; Le Novère et al., 2009; Touré et al., 2020; Schreiber et al., 2020; Waltemath et al., 2020), model databases (Glont et al., 2018; Malik-Sheriff et al., 2019), and standards for model annotation and documentation (Waltemath et al., 2011; Bergmann et al., 2014; Waltemath et al., 2020).

There is now also a journal specifically designed for replication studies of previous computational work (Hinsen & Rougier, 2019).

There are, however, persistent problems with model reproducibility. Not all modellers use available standards and share their code. Even for those who attempt to, there are often additional assumptions, e.g. about simulation parameters, model interfacing, or model data analysis that are not made explicit and that hinder future reproducibility (Waltemath et al., 2020).

To carefully annotate and document a model and ensure its reproducibility involves time and work. There is as yet little incentive or reward to doing this - the benefits of a model being reproducible often become clear years after it is first published, and scientific career paths are not currently structured to invite this level of foresight.

In theory, pre-publication peer review could pick up problems around reproducibility, but peer reviewers do not always have access to the code, computational resources, and time it would take to reproduce a model that they are reviewing. Post-publication review or replication studies (Hinsen & Rougier, 2019) are valuable, but may come too late to salvage the original model.

There is thus room for a new robust process to ascertain model reproducibility pre-publication, and even pre-submission, so that any reproducibility gaps can be caught and addressed early.

## Reproducibility Audits

In order to increase model reproducibility, we suggest the introduction of pre-publication reproducibility audits for modelling projects. This means that each project should involve a reproducibility auditor, whose role is to ensure the model is reproducible before it is published.

A reproducibility auditor would be a person familiar with the biological framework and modelling methodologies used, but who was not involved in the original model development. They would ideally not be part of the same research group as the original model developers, so that they do not share the workflows and implicit assumptions prevalent in that group.

When a computational model is ready for publication, the model developers send the model and write-up to their reproducibility auditor. The auditor attempts to run the model and reproduce the figures in the paper based on the information given to them, and identifies gaps in reproducibility. Both parties then work together to improve the documentation and ensure model reproducibility.

Once this is achieved, they submit the manuscript describing the model for publication together, with a short report of the reproducibility audit (steps taken, results obtained) in the appendix, and an author contribution statement specifying the role of the auditor.

The benefit for modellers is an external confirmation of reproducibility prior to publishing the model: possible gaps in documentation can thus be caught and fixed early.

For the field as such, the benefit is that there is an extra quality control step pre-publication. Journals could highlight this by introducing a “reproducibility audited” badge, similar to existing open science badges (Kidwell et al., 2016).

For the reproducibility auditor, the benefit is an opportunity to establish a collaboration with another research group in their field and learn first-hand about their modelling methods and process. This could be especially useful for early-career scientists just starting in a particular field. A simple reproducibility audit could even be an exercise assigned in a computational biology, biomedical informatics, or neuroscience class.

Standardisation efforts would benefit from reproducibility audits in two ways: First, reproducibility audits provide a good incentive for adhering to standards and good practices, thereby popularising the standards. Second, reproducibility audits will generate feedback on both the usefulness and usability of the standards used, and can thus feed back into standards development.

How will modellers find their reproducibility auditors? This could be done fairly informally within existing collaborative network. Some institutions or consortia may also create the role of a reproducibility auditor or make reproducibility audits part of the role of their research integrity advisors (Winchester, 2018).

For researchers not having access to these channels, there could also be a centralised online forum where people can post a short description of the project, techniques used, skills expected of auditor, expected workload associated with auditing and an indicative time frame. This may provide especially valuable opportunities for scientists who may not have access to mainstream scientific networks to act as auditors and thereby gain experience and establish collaborative ties.

## Turning Recommendations into Best Practice

How can both initiatives be incentivised, monitored and validated?

If a scientist invests time and resources in contributing to the experiment database, or serve as a reproducibility auditor, what is in it for them? We see several possibilities. The “microgrant” model of (small) cash incentives for solving particular problems is not entirely new. In data science, the Kaggle platform (https://www.kaggle.com/) challenges users to analyse data sets, sometimes (but not always) in competition for cash prizes. Over the last years, a wealth of interesting research papers have come out of Kaggle challenges. The potential for a similar crowd-sourcing approach to biomedical challenges has been recognised previously (Saez-Rodriguez et al., 2016). Additional incentives could be provided by opportunities for shared authorship and the establishment of new collaorations.

In the same way that Kaggle has been successfully used as a learning resource (Serrano et al., 2018), this is also a possibility here: Contributions to the incentivised experimental database or reproducibility audits could be a learning experience, for instance within the framework of an undergraduate assignment or Honours project. It could also be an opportunity for on-the-job learning for PhD students or postdoctoral researchers new to a particular field.

Our ideas fit within the bigger context of biocuration, in which datasets are structured, curated and annotated such that they adhere to FAIR-TLC principles (Howe et al., 2008; International Society for Biocuration, 2018). This movement has over the past decade or so striven to reframe data as an asset, that requires quality control and trust in those carrying this out (International Society for Biocuration, 2018; Gabrielsen, 2020).

Modellers arguably conduct biocuration in the course of the construction of their models. Parameters are chosen based on an expert assessment of the experiment that produced them, the use of these data are clearly defined, and there are standardised naming conventions for annotating model parts (Le Novère et al., 2005).

Our reproducibility audits therefore fall under the quality control aspect of curation, and can take much from recent guidance in this area (Tang et al., 2019). Here again, the idea of using curation as a teaching tool has already been brought up: Undergraduates have been shown to be just as capable at biocuration as experts after training (Mitchell et al., 2015), and from our own experience quickly acquire the proficiency required to critically evaluate model data inputs and outputs. This, if combined with the experimental database idea, could harness student expertise in driving model validation, expose potential researchers early in their career to FAIR principles, and introduce modelling methods as a way of structuring existing data into valuable and usable formats.

Ultimately, the ideas laid out here have to be tested and evaluated. If an incentivised experimental database with an attached microgrant scheme were to be implemented, it would make sense to monitor not only application and success rates, but also completion of projects, data submission, and subsequent use, for instance in models and follow-up publications.

The use of reproducibility audits could easily be tracked if journals were to introduce a “reproducibility audited” badge. This would also allow an analysis of the impact (e.g. model downloads from repositories or paper citations) of audited vs non-audited models.

## Conclusions

We show here that the fostering of collaborative practices has potential for improving the validity of biochemical models, both external and internal. These collaborations are designed to be mutually beneficial, bringing researchers in similar fields closer together whilst also addressing the challenge of model validity. Furthermore, they have the potential to both accelerate model development and increase the number of biologically-derived parameters available to all researchers. The specifics of how to exactly implement these ideas should also be collaboratively decided. By setting out a possible framework we now invite readers to discuss and debate how we can best turn these ideas into reality.

## Information Sharing Statement

This article is a think piece, for which no data was produced or analyzed. Readers who nonetheless want to access the data can do so here: http://tinyurl.com/nein2022data.
